# A Return of the Sick of the Ship's Company, and of the Military, on Board the Ships in the Service of the Honourable the United East-India Company, for the Years 1792 and 1793
*In this Table the number of sick sometimes exceeds the number of the ship's company. If the same man is two or three times sick during the voyage, and recovers, this must be the case. Sometimes also the number of recovered and dead are not equal to the number of sick; but this must also happen when, at the end of the voyage, some still remain on the sick list.


**Published:** 1795

**Authors:** John Lorimer


XII. *Return* of the Ship's Company, and of the Military, on Board the Ships in the Service of the Honourable the United Eaſt-India Company, for the Years 1792 and 1793.* *By* John Lorimer, *M. D.*

| Ships' Names. | Deſtination. | Ships' Company outward. | Sick. | Recovered. | Dead | Ships' Company homeward. | Sick. | Recovered. | Dead | In Port. | Sick. | Recovered. | Dead | Number of Recruits outward | Sick. | Recovered. | Dead | Invalids, &c. homeward. | Sick. | Recovered. | Dead |
|---|---|---|---|---|---|---|---|---|---|---|---|---|---|---|---|---|---|---|---|---|---|
| Ocean | St. Helena, Madras, and China | 134 | 102 | 100 | 1 | 124 | 45 | 45 | 0 | St. Helena, Madras, and China | 77 | 74 | 3 | 309 | 62 | 61 | 1 | 8 | 1 | 1 | 0 |
| Nottingham | Coaſt and China | 128 | 67 | 65 | 2 | 112 | 12 | 12 | 0 | Coaſt and China | 54 | 44 | 10 | 218 | 109 | 106 | 3 | 52 | 0 | 0 | 0 |
| Lord Macartney | Ditto | 105 | 49 | 49 | 0 | 105 | 22 | 21 | 1 | Ditto | 80 | 78 | 2 | 181 | 144 | 140 | 4 | 21 | 8 | 8 | 0 |
| Ganges | Ditto | 106 | 40 | 33 | 7 | 104 | 14 | 14 | 0 | Ditto | 25 | 24 | 1 | 163 | 69 | 64 | 5 | 70 | 0 | 0 | 0 |
| Sir Edward Hughes | Ditto | No Regular Return out. | | | | 110 | 56 | 53 | 3 | Ditto | No Return. | | | No Return. | | | | 5 | 0 | 0 | 0 |
| Europa | Ditto | 103 | 86 | 85 | 1 | 106 | 136 | 132 | 4 | Ditto | 148 | 126 | 5 | 428 | 264 | 263 | 1 | 60 | 45 | 42 | 3 |
| Melville Caſtle | Coaſt and Bay | 106 | 15 | 13 | 1 | 103 | 25 | 22 | 3 | Coaſt and Bay | 38 | 30 | 5 | 387 | 125 | 119 | 1 | 39 | 12 | 10 | 3 |
| Contractor | Ditto | 105 | 53 | 53 | 0 | 100 | 50 | 47 | 3 | Ditto | 89 | 82 | 7 | 193 | 186 | 183 | 3 | 82 | 53 | 48 | 5 |
| Ponſborne | Ditto | 106 | 47 | 45 | 0 | 99 | 12 | 10 | 0 | Ditto | 59 | 58 | 1 | 157 | 22 | 20 | 2 | 98 | 5 | 4 | 1 |
| Buſbridge | Ditto | 106 | 56 | 55 | 1 | 94 | 12 | 11 | 1 | Ditto | 30 | 26 | 4 | 138 | 74 | 72 | 2 | 46 | 2 | 2 | 0 |
| Roſe | Ditto | 104 | 25 | 25 | 0 | 121 | 42 | 40 | 2 | Ditto | 39 | 37 | 2 | 142 | 35 | 32 | 3 | 88 | 13 | 11 | 2 |
| King George | Ditto | 99 | 19 | 19 | 0 | 120 | 25 | 23 | 2 | Ditto | 26 | 24 | 2 | 155 | 26 | 24 | 2 | 69 | 13 | 11 | 2 |
| Rockingham | Bombay and China | 106 | 42 | 41 | 1 | 101 | 9 | 8 | 1 | Bombay and China | 58 | 55 | 3 | 168 | 68 | 67 | 1 | 25 | 0 | 0 | 0 |
| Sulivan | Ditto | 113 | 51 | 49 | 0 | 103 | 94 | 93 | 1 | Ditto | 89 | 82 | 7 | 95 | 39 | 39 | 0 | 10 | 3 | 3 | 0 |
| Middleſex | Ditto | 111 | 20 | 15 | 5 | 107 | 10 | 9 | 1 | Ditto | 35 | 31 | 4 | 160 | 80 | 76 | 4 | 6 | 2 | 2 | 0 |
| Duke of Montroſe | Bombay | 101 | 46 | 46 | 0 | 101 | 31 | 31 | 0 | Bombay | 61 | 61 | 0 | 186 | 60 | 59 | 1 | 76 | 18 | 18 | 0 |
| General Elliot | Ditto | 105 | 16 | 16 | 0 | 105 | 29 | 26 | 3 | Ditto | 47 | 43 | 4 | 176 | 26 | 24 | 2 | 61 | 3 | 1 | 2 |
| Earl of Wycombe | Bencoolen and China | 87 | 83 | 82 | 1 | 82 | 27 | 24 | 3 | Bencoolen and China | 20 | 20 | 0 | 8 | 0 | 0 | 0 | 1 | 0 | 0 | 0 |
| Valentine | St. Helena, Bengal, and Bencoolen | 106 | 19 | 19 | 0 | 97 | 30 | 27 | 3 | St. Helena, Bengal, and Bencoolen | 86 | 84 | 2 | 73 | 23 | 23 | 0 | 96 | 50 | 45 | 5 |
| General Goddard | Bengal | 106 | 27 | 26 | 1 | 104 | 31 | 29 | 2 | Bengal | 71 | 61 | 10 | 155 | 110 | 109 | 1 | 64 | 14 | 14 | 0 |
| Bellmont | Ditto | 90 | 110 | 110 | 0 | 85 | 65 | 60 | 5 | Ditto | 106 | 89 | 11 | 17 | 2 | 2 | 0 | 52 | 30 | 29 | 1 |
| Earl Talbot | Ditto | 90 | 40 | 36 | 4 | 78 | 37 | 36 | 1 | Ditto | 27 | 25 | 2 | 15 | 2 | 1 | 1 | 28 | 10 | 7 | 3 |
| Laſcelles | China | 108 | 67 | 67 | 0 | 108 | 11 | 8 | 3 | China | 116 | 110 | 6 | 4 | 0 | 0 | 0 | 7 | 0 | 0 | 0 |
| Walpole | Ditto | 105 | 53 | 52 | 1 | 107 | 43 | 41 | 2 | Ditto | 73 | 70 | 3 | 0 | 0 | 0 | 0 | 2 | 0 | 0 | 0 |
| Thetis | Ditto | 106 | 95 | 94 | 1 | 105 | 136 | 132 | 4 | Ditto | 75 | 73 | 2 | 9 | 0 | 0 | 0 | 6 | 0 | 0 | 0 |
| Royal Admiral | Port Jackſon and China | 121 | 25 | 24 | 1 | 120 | 54 | 33 | 3 | Port Jackſon and China | 4 | 1 | 5 | Convicts 392 | 225 | 139 | 13 | 3 | 0 | 0 | 0 |
| | | 2657 | 1253 | 1219 | 28 | 2701 | 1058 | 987 | 51 | | 1533 | 1408 | 96 | 3929 | 1751 | 1623 | 50 | 1075 | 282 | 256 | 27 |

Dublin, Airly Caſtle, Bridgewater } Of theſe three Ships, which failed in the Year 1791, there are no returns.

* In this Table the number of ſick ſometimes exceeds the number of the ſhip's company. If the ſame man is two or three times ſick during the voyage, and recovers, this muſt be the caſe. Sometimes alſo the number of recovered and dead are not equal to the number of ſick; but this muſt alſo happen when, at the end of the voyage, ſome ſtill remain on the ſick liſt.

XIII. *An*

